# Acute hearing and visual loss caused by thiamine deficiency

**DOI:** 10.1186/s12883-023-03337-w

**Published:** 2023-07-31

**Authors:** Junrong Xu, Fei Li, Yongjie Xue

**Affiliations:** 1 Department of Gastroenterology, The Affiliated Hospital of Northwest University, Xi’an No.3 Hospital, Xi’an, Shaanxi 710018 People’s Republic of China; 2 Department of Radiology, The Affiliated Hospital of Northwest University, Xi’an No.3 Hospital, Xi’an, Shaanxi 710018 People’s Republic of China

**Keywords:** Wernicke encephalopathy, Liver cirrhosis, Bilateral blindness, Bilateral hypoacousia

## Abstract

**Background:**

Wernicke encephalopathy (WE) is a devastating acute or subacute neurological disorder caused by thiamine deficiency. Wernicke encephalopathy is characterized by the triad of ocular signs, cerebellar dysfunction, and confusion. Visual loss and hearing loss are less common findings in WE. Here, we report a case of Wernicke encephalopathy in a nonalcoholic liver cirrhosis patient who presented with acute bilateral deafness and bilateral blindness.

**Case presentation:**

A 60-year-old Chinese man presented with a history of bilateral blindness and bilateral hypoacousia for 3 days. He had a history of liver cirrhosis and chronic hepatitis C virus infection and did not have a habit of alcohol consumption. Ophthalmologic and otologic examinations showed no obvious abnormalities. MRI findings revealed symmetric fluid-attenuated inversion recovery (FLAIR) hyperintensities in the bilateral medial dorsal thalamus, periventricular region around the third ventricle and tectum, and dorsal medulla oblongata. One day after hospitalization, the patient developed a mild coma. Based on the laboratory and neuroimaging findings, we diagnosed the patient with Wernicke encephalopathy. He soon regained consciousness after administration of thiamine. Both his visual acuity and his hearing function improved gradually.

**Conclusions:**

We suggest that Wernicke encephalopathy can present with bilateral blindness and bilateral deafness.

## Background

Wernicke encephalopathy (WE) is a devastating acute or subacute neurological disorder caused by thiamine (vitamin B1) deficiency. Wernicke encephalopathy is most commonly seen in chronic alcohol abusers but is rare in patients with nonalcoholic liver disease. Wernicke encephalopathy is characterized by the triad of ocular signs, cerebellar dysfunction, and confusion [1]. In the literature, visual loss and hearing loss are rarely described as the first manifestations of WE [2–4]. We report a case of Wernicke encephalopathy in a nonalcoholic liver cirrhosis patient who presented with acute bilateral deafness and bilateral blindness.

### Case presentation

**Figure 1:**
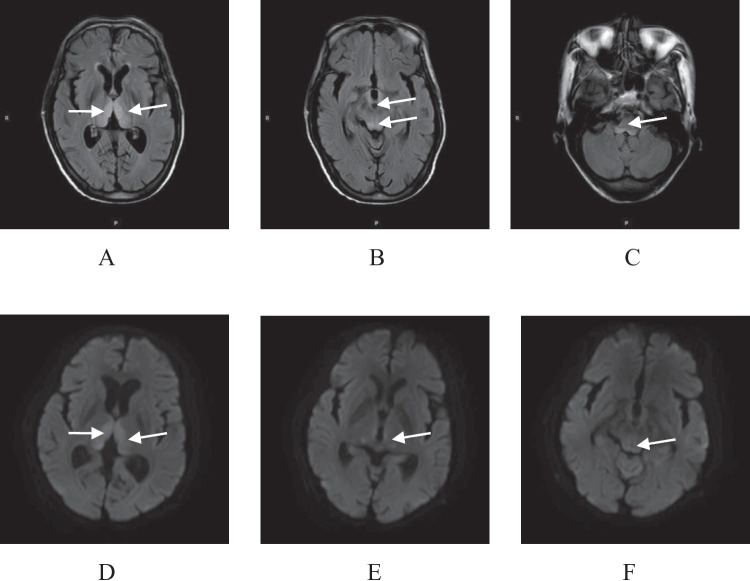
**Fig. 1** MRI of the brain. T2-FLAIR imaging demonstrated symmetric hyperintensities (arrows) in the bilateral medial dorsal thalamus (**A**), periventricular region around the third ventricle and tectum (**B**), and dorsal medulla oblongata (**C**). DWI demonstrated symmetric hyperintensities (arrows) in the bilateral medial dorsal thalamus (D), geniculate body (E), and the region around the periaqueductal grey matter (**F**)

A 60-year-old Chinese man presented with a history of bilateral blindness and bilateral hypoacousia for 3 days. He had a history of liver cirrhosis and chronic hepatitis C virus infection and did not have a habit of alcohol consumption. Two weeks earlier, the patient experienced abdominal distension, nausea, and vomiting because of a lack of passage of stools. Following therapeutic fasting for 10 days, he developed bilateral blindness and bilateral hypoacousia. At the same time, his son noticed signs of mild disorientation, impaired memory, and slurring of speech. His laboratory indices were as follows: blood total bilirubin 64.6 μmol/L (normal level, ≤ 26), direct bilirubin 29.4 μmol/L (≤ 4), albumin 27.6 g/L (40–55), alanine aminotransferase 24 U/L (9–50), aspartate aminotransferase 33 U/L (15–40), international normalized ratio (INR) 1.3 (0.9–1.2), ammonia 57.86 μmol/L (18 –32), serum sodium 122.89 mmol/L (137–147), serum chloride 95.75 mmol/L (99–110),and blood glucose 5.7 mg/dL (3.2–6.0). Ophthalmic examination revealed a visual acuity of slight light perception in both eyes, and other examinations demonstrated no evidence of fundus haemorrhage, papilledema, retinal detachment, or abnormal colour recognition. The tuning fork test showed no significant abnormalities. However, the patient refused to undergo visual and auditory evoked potential testing. Magnetic resonance imaging (MRI)T2-FLAIR imaging demonstrated symmetric hyperintensities in the bilateral medial dorsal thalamus, periventricular region around the third ventricle and tectum, and dorsal medulla oblongata. Diffusion-weighted imaging (DWI) demonstrated symmetric hyperintensities in the bilateral medial dorsal thalamus, geniculate body, and the region around the periaqueductal grey matter (Fig. 1).After admission, the patient was treated with intravenous sodium chloride and ornithine aspartate. Simultaneously, oral lactulose was administered to the patient.

Subsequently, the plasma ammonium level returned to a normal level and was accompanied by an increase in serum sodium to 130 mmol/L. However, the patient’s clinical symptoms worsened into a slight coma rather than improving. He was immediately administered 100 mg of intravenous thiamine owing to the high suspicion index for Wernicke encephalopathy The patient soon regained consciousness but still had blindness and deafness. Therefore, he was administered intravenous vitamin B1 (100 mg daily) for the next 7 days. After the administration of thiamine, both his visual acuity and his hearing function improved gradually.

## Discussion and conclusions

WE is a devastating acute or subacute neurological disorder caused by thiamine (vitamin B1) deficiency. Although it is most commonly seen in chronic alcohol abusers, it can occur in any disorder associated with malnutrition, including severe anorexia, hyperemesis gravidarum, malignancy, fasting, or small bowel obstruction. WE is rare in patients with nonalcoholic fatty liver disease. This patient with nonalcoholic liver cirrhosis developed WE after therapeutic fasting for ten days because of ileus. Thiamine is a water-soluble essential vitamin obtained from the diet. It is essential for the maintenance of the osmotic gradient in the cell membrane, glucose metabolism, and neurotransmitter synthesis. Thiamine deficiency results in a reduced osmotic gradient, causing swelling of intracellular spaces, especially in the periventricular regions with a high rate of thiamine-related glucose and oxidative metabolism [1].

WE is characterized by the triad of ocular signs, cerebellar dysfunction, and confusion.The clinical diagnosis of WE requires two of the following four criteria: (i) dietary deficiencies; (ii) ocular signs; (iii) cerebellar dysfunction; and (iv)altered mental state or mild memory impairment [5].This patient had a history of dietary deficiencies, altered sensorium, and memory impairment. He regained consciousness soon after the administration of thiamine.Therefore, he met the diagnostic criteria for WE. The unique feature in this case was the sudden onset of bilateral blindness and bilateral deafness during the initial phase of the disease.

Visual loss and hearing loss are less common findings in WE. Visual loss in WE results from a metabolic aetiology related to bilateral optic disc swelling or impairment of visual pathways [6]. Bilateral blindness has been observed to occur in WE, while bilateral deafness has rarely been reported. The pathogenesis of hearing loss in patients with WE is not clear. It is assumed to be due to the impairment of geniculate nuclei of the thalamus and peripheral damage to the acoustic nerves because of thiamine deficiency [7].Cases of WE presenting with bilateral blindness and bilateral deafness are fairly rare. Moussa et al. [3] reported a case of a chronically vomiting man with bilateral blindness that was associated with bilateral perceptual hypoacousia, revealing the presence of WE. However, the mechanisms underlying this patient's bilateral blindness and bilateral deafness were not elucidated. In some inherited thiamine-related diseases, hearing loss is a prominent feature, and optic atrophy has been described. The pathogenesis of visual and hearing impairment caused by thiamine transport and metabolism gene defects has been clearly studied. Thiamine-responsive megaloblastic anaemia (TRMA) is an autosomal recessive disease whereby active thiamine uptake into cells is disturbed [8].This disorder has been linked to mutations in the SLC19A2 gene, which encodes a functional thiamine transporter. Thiamine transporter gene defects lead to dysfunction of multiple cellular metabolic processes. TRMA is characterized by megaloblastic anaemia, diabetes, and sensory-neural deafness and is also associated with congenital heart disease, arrhythmia, cardiomyopathy, retinal degeneration, optic atrophy, and stroke [8, 9].

The etilology of the bilateral blindness and bilateral deafness in this patient is not clear. In this patient, DWI restriction was observed in the medial and lateral geniculate bodies, indicating impairment to visual and auditory pathways. It is unclear what provoked this patient's bilateral blindness and bilateral deafness. It is plausible that the blindness and deafness of this patient may have been caused by a thiamine deficiency that resulted in aberrant cellular metabolic processes in the visual and auditory pathways, similar to the hearing and vision loss brought on by TRMA. However, due to the patient's transient thiamine deficiency, no significant abnormalities were found in the relevant ophthalmologic and otologic examinations.

Hyponatremic and hepatic encephalopathy are common causes of metabolic encephalopathy that may coexist in patients with cirrhosis [10]. Acute bilateral loss of vision caused by cortical blindness(CB)may occur in patients exhibiting hepatic encephalopathy. CB refers to visual loss due to bilateral lesions of the visual pathways in the temporo-occiptal lobes, with normal pupillary light reflexes and normal findings [11]. In hepatic encephalopathy, metabolic disturbance can lead to CB. Most hepatic CB patients who undergo MRI scanning show no abnormal findings [12].Although this patient presented high levels of blood ammonium, there were no other symptoms of hepatic encephalopathy. After receiving oral lactulose therapy, his blood ammonia dropped, but the patient's symptoms continued to worsen. Moreover, head MRI showed typical features of Wernicke's encephalopathy. Therefore, his visual impairment was not attributed to the high levels of blood ammonia or hepatic encephalopathy, which demonstrates a rare case of Wernicke's encephalopathy.

Hyponatremia presenting with visual disturbance has been described in a few patients [13], but bilateral deafness has not been reported in patients with hyponatremia. Despite having hyponatremia before admission, the patient showed no signs of recovery following sodium chloride therapy. The patient regained consciousness upon thiamine administration, and both his visual acuity and his hearing function improved gradually. We therefore believe that this patient's symptoms were caused by Wernicke's encephalopathy.

MRI is a reliable test for WE. The reported sensitivity and specificity of MRI for the diagnosis of WE are 53% and 93%, respectively [14]. Increased T2-weighted and FLAIR signal intensities in the periaqueduct, periventricular, and medial thalami regions are the characteristic features [5]. This patient showed typical MRI findings of symmetric FLAIR hyperintensities in the bilateral medial dorsal thalamus, periventricular region around the third ventricle and tectum, and dorsal medulla oblongata. The MRI findings coupled with the clinical presentation are consistent with acute WE. Hyperintense signals on DWI can be due to restricted water diffusion in the lesion [15]. In this patient, DWI restriction was observed in the medial and lateral geniculate bodies, indicating impairment of the visual and auditory pathways. Some studies have demonstrated that MRI has a high specificity but low sensitivity for WE [14, 16]. However, a large number of researchers believe that MRI is a powerful and reliable tool that should be used to support the diagnosis of acute WE in both alcoholic and and nonalcoholic individuals [5, 17, 18].

Thiamine or erythrocyte nonalcoholic individuals transketolase blood levels are very important to the diagnosis of WE. Whenever WE is suspected, a blood sample for measurement of total thiamine should be drawn immediately before administration of thiamine [5]. Due to a lack of appropriate testing equipment, this patient unfortunately did not undergo serum thiamine or erythrocyte transketolase blood level testing. However, based on the clinical evidence of a nutritional deficiency, the patient's acute presentation, the patient's response to thiamine therapy, and the results of MRI, the diagnostic criteria for WE in this patient were satisfied.

## Abbreviations

WE: Wernicke encephalopathy
FLAIR: Fluid-attenuated inversion recovery
MRI: Magnetic resonance imaging
DWI: Diffusion-weighted imaging
CB: Cortical blindness
TRMA: Thiamine-responsive megaloblastic anaemia
